# Evolving Antibiotics against Resistance: a Potential Platform for Natural Product Development?

**DOI:** 10.1128/mBio.02946-19

**Published:** 2019-12-24

**Authors:** Kristofer Wollein Waldetoft, James Gurney, Joseph Lachance, Paul A. Hoskisson, Sam P. Brown

**Affiliations:** a School of Biological Sciences, Georgia Institute of Technology, Atlanta, Georgia, USA; b Strathclyde Institute of Pharmacy and Biomedical Sciences, University of Strathclyde, Glasgow, United Kingdom; McMaster University

**Keywords:** actinomycetes, antibiotic, evolution, experimental evolution, natural antimicrobial products

## Abstract

To avoid an antibiotic resistance crisis, we need to develop antibiotics at a pace that matches the rate of evolution of resistance. However, the complex functions performed by antibiotics—combining, e.g., penetration of membranes, counteraction of resistance mechanisms, and interaction with molecular targets—have proven hard to achieve with current methods for drug development, including target-based screening and rational design. Here, we argue that we can meet the evolution of resistance in the clinic with evolution of antibiotics in the laboratory. On the basis of the results of experimental evolution studies of microbes in general and antibiotic production in *Actinobacteria* in particular, we propose methodology for evolving antibiotics to circumvent mechanisms of resistance. This exploits the ability of evolution to find solutions to complex problems without a need for design. We review evolutionary theory critical to this approach and argue that it is feasible and has important advantages over current methods for antibiotic discovery.

## Abstract (precis)

To avoid an antibiotic resistance crisis, we need to develop antibiotics at a pace that matches the rate of evolution of resistance. However, the complex functions performed by antibiotics—combining, e.g., penetration of membranes, counteraction of resistance mechanisms, and interaction with molecular targets—have proven hard to achieve with current methods for drug development, including target-based screening and rational design.

## OPINION/HYPOTHESIS

The evolution of antibiotic resistance is a major threat to human health, and key to meeting this challenge is the development of novel drugs (1). Despite early successes during the “golden era” (2) of antibiotic discovery, however, drug development is currently struggling to replenish the antibiotic arsenal at a sufficient rate to maintain adequate treatment options. Natural product discovery programs that formed the basis of the “golden era” were cancelled due to diminishing returns and problems with dereplication, yet the programs that succeeded them, including high-throughput target-based screening of synthetic compound libraries and rational drug design, have yielded little (2). The shortcomings of these latter approaches have, in turn, resulted in renewed interest in natural products and novel methods for their discovery (3–6).

However, while the modernized search for natural product antibiotics does have potential (6), all methods for natural product discovery have a fundamental limitation built into them: they are confined to compounds that have already evolved. We argue that this limitation can be removed. That is, rather than merely discovering natural product antibiotics, we can take control of the process that gives rise to them; we can bring antibiotic evolution into the laboratory, and control it, to evolve the compounds that we need. Though not an established method for drug development, experimental evolution is a routine tool in evolutionary biology, and it has been successfully applied to antibiotic production by *Actinobacteria* (7), the source of the majority of antibiotic classes in clinical use.

**FIG 1:**
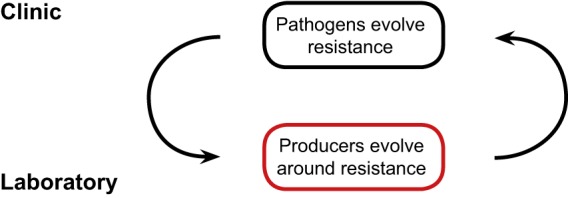
Proposed roles of evolution in the arms race between pathogens and drug development. An evolutionary arms race between pathogens and drug development is illustrated. In clinical use, the antibiotic is held constant, whereas pathogens respond by evolving resistance. Experimental evolution of antibiotic-producing organisms is then used to modify the antibiotic or discover other compounds, using resistant bacteria to impose selection. The novel or modified drug succeeds or complements the original drug in clinical use.

In brief, we propose the following: when the utility of natural product antibiotics is threatened by resistance, we can experimentally evolve organisms that produce these compounds to circumvent the resistance mechanisms. This evolution may modify the compounds to counteract how resistance is achieved (e.g., decrease the sensitivity of β-lactams to degradation by β-lactamases) or upregulate previously silent pathways to produce novel antibiotics (7) or antiresistance molecules (e.g., β-lactamase inhibitors). An overview of the approach is given in Fig. 1.

## WHY EVOLVE ANTIBIOTICS?

The starting point for the argument is a simple observation: it is evolution that has the track record in antibiotic development. Despite extensive efforts with synthetic chemistry approaches, most antibiotics in the clinic either are natural products or are derived from such products (3), that is, they are ultimately the result of evolution. It is thus unquestionable that evolution is apt to produce antibiotics. The question is “Can adequate evolution be achieved in the laboratory on a reasonable timescale?" Next, we argue that it can.

### Experimental evolution of antibiotics is feasible.

First, natural product antibiotics may be particularly well suited to evolutionary discovery and modification. The potential for discovery of novel compounds is indicated by the fact that *Actinobacteria*—the antibiotic-producing bacteria that formed the basis of the “golden era” (2)—carry genes that encode many cryptic pathways (2, 8) that experimental evolution may turn on (7). There is also potential for modification of existing compounds. Consider the cephalosporins, a key class of antibacterial drugs. These drugs consist of a core chemical scaffold decorated with side chains, where the latter often modulate pharmacological properties. Most notably, the R1 side chain affects the susceptibility of the compounds to degradation by β-lactamases, as well as their affinity for penicillin binding proteins (9). This has formed a basis for semisynthetic chemistry to improve these drugs (9) and can provide scope for evolutionary adaptation. A similar pattern with variations on a common core is seen in the aminoglycosides, where it has likely facilitated evolution (10). Being synthesized and decorated by multipart pathways with many enzymatic steps, natural product antibiotics can evolve by mutation, acquisition, or even loss of enzymes that determine their final structure. In addition, evolution can interconnect different pathways to produce novel compounds by combinatorial biosynthesis, as was recently reported for naturally evolved systems (11, 12). It is plausible that the genes and gene clusters that encode components of biosynthetic pathways are conducive to evolution, because their modular organization facilitates rearrangement and other modification of genetic material (10). These routes to compound improvement are a mere illustration of expected evolutionary potential, however; a key strength of the evolutionary process is precisely its ability to produce unanticipated results by probing chemical space independently of prior knowledge and the toolkit of synthetic chemistry.

Second, in a landmark study, Charusanti et al. used 4 months of experimental evolution of Streptomyces clavuligerus in coculture with methicillin-resistant Staphylococcus aureus to turn on a previously silent pathway for an antibiotic that inhibited the growth of the target pathogen (7). This shows that adequate selection can be imposed in the laboratory setting, that the producer organism can respond to such selection on a practically useful timescale, and that upregulation of a silent biosynthetic pathway is within the scope of experimental evolution. These results hold great promise, because the development and optimization of methods to switch on silent pathways would provide access to a vast untapped source of novel antibiotics (13). Beyond this empirically known potential, we propose that experimental evolution can also mutate and recombine biosynthetic gene clusters to modify compounds to perform their functions better or upregulate several compounds that work in concert (e.g., antibiotics and antiresistance compounds).

### Experimental evolution has advantages over target-based screening and rational design.

Natural evolution has been very successful in developing antibiotic compounds. The discoveries during the “golden era” are a result of natural evolution, and current trends toward revival of natural product discovery (see above) take it for granted (and for good reason—the genus *Streptomyces* alone is estimated to be able to produce more than 100,000 antimicrobial compounds [14], only a minute fraction of which are in clinical use). We argue that there is reason to believe that the evolution of producer organisms has an edge over current approaches to antibiotic development.

The function that an antibiotic has to perform—to kill or inhibit the target bacterium—may seem simple, but in fact, it is complex and consists of several components. These include the penetration of membranes, circumvention of resistance mechanisms, and binding to target molecules, as relevant. As a consequence, useful molecules are hard to identify by high-throughput screens for individual component functions or to rationally design (2, 4). In addition, nonpeptide antibiotics are difficult targets for so-called directed-evolution approaches, which are currently best developed for peptides and proteins (15).

Experimental evolution, by contrast, like its natural counterpart, applies selection on the producer organism as a whole and can thereby modify not only a single protein, gene, or pathway but an entire metabolic system. It is therefore better suited for small-molecule development than is the directed-evolution methodology. Moreover, and more importantly, by applying selection for a complex function on a rich metabolic system, experimental evolution takes advantage of a key aspect of adaptive evolution: selection for a complex function (e.g., target killing) entails selection for all its necessary component functions (e.g., membrane penetration and resistance circumvention, as relevant). This ability of evolution to subsume the optimization of individual mechanisms under a higher-level function makes it possible to define the goal of the evolutionary process and apply selection for attaining it, without explicitly addressing the chemical properties required. In the case considered here, this goal is the killing of a resistant target bacterium, ideally under physiologically realistic conditions. In Box 2 (point 10 in particular), we discuss problems that may arise when translating this principle into practice.

## HOW TO EVOLVE ANTIBIOTICS

**FIG 2:**
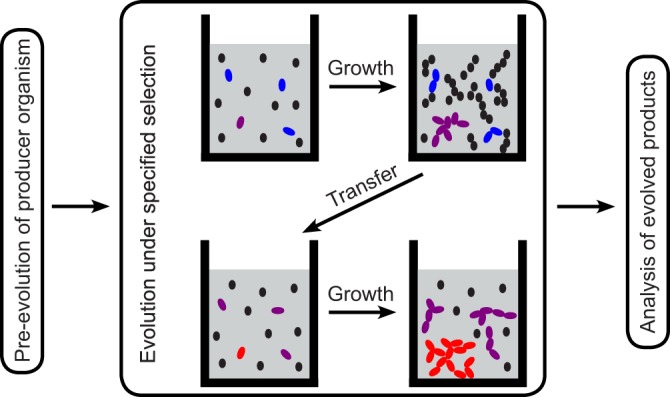
Experimental evolution procedure. Two growth cycles of a serial passage are represented. The producer (colored) competes with the target (black) in a structured medium. A mutant producer (purple) has a competitive advantage due to an improved compound. An additional mutation (red) further improves the compound, etc. As a result, the killing efficiency increases, and the target density decreases over passages. The target’s density and degree of resistance can be adjusted to compensate.

The centerpiece of the approach is to evolve antibiotic-producing organisms around resistance mechanisms by applying the appropriate selection pressure in the laboratory. The principal method is experimental evolution by serial passage in coculture, where the antibiotic producer is subjected to antagonistic interaction (for example, competition over nutrients) with a target organism that is resistant to its antibiotic, as detailed in Box 1 and represented in Fig. 2.

In general, experimental evolution by serial passage is designed to allow adaptations to evolve gradually, through a sequence of genetic changes, over many passages and bacterial generations, and thereby create novel genotypes that are substantially different from the genotype of the ancestral organism with which the procedure was started. In the specific application we propose, the idea is to use the target organism to impose selection pressure on the producer to evolve counteradaptations to the target’s resistance.

We advocate antagonistic interactions with resistant organisms to impose selection on the producer, rather than manually selecting colonies based on their killing zones (a strategy with which it can in principle be combined). The reason is that antagonistic interactions can select on individual cells or small cell clusters. This increases the number of units of selection, a key parameter in experimental evolution. It also makes it possible to run the experiment in smaller volumes that are more conducive to high-throughput methodologies. We anticipate that a 96-well format will yield producer population sizes on the order of 10^6^ cells. Because the efficacy of natural selection depends on the product of effective population size and the magnitude of selection coefficients (16), this will enable selection of even weakly beneficial alleles. Evolution requires the presence of genetic variation, and mutation rates in producers may be increased by the use of mutator strains or mutagens (see Box 2).

Experimental evolution of microorganisms is well established (17), and much of the groundwork for what we advocate here has, consequently, been done. In the following, we discuss evolutionary theory of key importance to its application to antibiotic development. We keep the main text conceptual. In Box 1, we illustrate the experimental evolution procedure with a protocol skeleton, and in Box 2, we address potential problems.

### Choosing producer and target organisms.

The natural choice of producer organism are bacteria that are known to have a high potential for natural product biosynthesis. Most notable in this regard are the *Actinobacteria*. These bacteria were the mainstay of the “golden era” (2). They are a focus of recent efforts to revive natural product discovery (8), and they have been successfully used for experimental evolution of antibiotic production through competition with resistant target bacteria (7). In addition, even well-studied *Actinobacteria* have cryptic pathways (2, 8) that experimental evolution can activate (7). This variety of pathways is also key to the evolutionary modification of antibiotics; it provides scope for mutations that regulate, modify, or hybridize different pathways to catalyze the reactions needed to improve the original compound. If the genomes of single strains should not suffice to supply the necessary genetic material and diversity for selection to act upon, the observation that biosynthetic gene clusters are well adapted for horizontal transfer (18) suggests that the genetic substrate can be extended to ensembles of strains or species. This could, in principle, be achieved by artificial introduction of gene clusters into producer strains or by coculture of genetically diverse producer bacteria.

While a genome (or population of genomes) possessing a rich set of biosynthetic gene clusters provides evolutionary substrates for the evolution of pathways to new compounds, this process may be constrained by the need for producer bacteria to survive any new antibiotics that are produced. If, for example, the mechanism by which the producer bacterium resists its compound is similar to the resistance mechanism of the target bacterium, evolutionary changes that circumvent target resistance may be suicidal for the producer. Adaptive evolution of the antibiotic due to selection for target killing may then require simultaneous evolution of resistance in the producer. This requirement is double edged: it limits the rate at which successful mutants arise, because complementary mutations are needed in two systems, but once such mutants are present, it also increases the efficiency of selection, since the mutant would kill not only target bacteria but other producers as well. The problem may be avoided by choosing producer and target bacteria with different mechanisms of resistance, so that counteraction of target resistance is unlikely to interfere with producer resistance.

This issue also highlights that the optimal choice of producer organism depends on the intended evolutionary outcome. If the goal is to discover compounds that have already evolved in nature by turning on preexisting, but silent, pathways, a relatively uncharacterized strain with a largely unexplored biosynthetic capacity seems best. If, on the other hand, one aims to modify an antibiotic to avoid a resistance mechanism, knowledge of the resistance mechanisms of both the target and producer is advantageous, and a better-characterized producer strain may be preferable.

In addition to the *Actinobacteria*, on which we focus, *Gamma*- and Deltaproteobacteria may also be used as producers. For example, antibiotic-producing predatory myxobacteria could be evolved to feed on resistant prey. (See references 19 and 20 for reviews of antibiotics and other natural products in myxobacteria.)

The function of the target organism is to impose selection on the producer to kill it. It should therefore interact antagonistically with the producer. The interaction may be competition over a limiting resource or predation, where the producer organism gains access to nutrients by lysing target cells. The results of Charusanti et al. (7), discussed above, show that a resistant human pathogen can be used, but it is also possible to express the resistance mechanism of interest in a vehicle organism. There are pros and cons to both alternatives. The use of resistant human pathogens promotes medical relevance, especially for evolutionary responses that do not directly interfere with the resistance mechanism, but result in the killing of the target some other way, whereas the cloning of resistance genes allows one to choose a vehicle organism that fits the precise protocol to be used in the experimental evolution process. Pertaining to the latter aspect are the ability to interact with the producer and to be easily separable from it in serial transfers, so as to avoid evolution of the target.

### Antibiotics are public goods.

Adaptive evolution requires that the benefit conferred by a mutation be enjoyed by individuals that carry that mutation rather than individuals that do not. In many cases, this is not problematic. If, for example, a population of bacteria is exposed to an antibiotic, and in one of the cells, a mutation arises that decreases its susceptibility to the antibiotic, that cell will enjoy a benefit. If, on the other hand, bacteria produce an antibiotic that kills competitors, and one producer cell has a mutation that improves the potency of the antibiotic and thereby reduces competition, selection does not necessarily favor that cell. The reason is that the antibiotic is secreted into the environment, where it spreads by diffusion and mass flow, to the benefit of all cells in the vicinity, regardless of whether they carry the mutation or not. That is, antibiotics are not private, but public, goods.

In order to evolve antibiotics and similar compounds, it is therefore necessary to devise a way to link the benefit associated with an improved version of a compound (or the production of a novel compound) to cells that carry the underlying genotype. Fortunately, the evolution of public goods has been extensively studied, and several routes have been found (21–26). Here, we focus on the one we think is most practically useful: spatial structure.

The key to spatial structure is the restriction of movement of cells and the molecules that they produce. Since cells reproduce by division, population growth in structured environments gives rise to clusters of cells that belong to the same clone. Due to their clonality, the cells within a cluster tend to have, or lack, the same mutations, and benefits conferred by such mutations will be preferentially enjoyed by cells that carry those mutations, simply because cells with the same genotype are closer to each other and to their products than are cells of different clones. In more general terms, the variation in the compounds produced, and the underlying genotypes should be spatially structured. The degree of structure that is achieved depends on several factors, including the medium used and the producer’s mode of growth. For example, the filamentous growth of *Actinobacteria* may tend to mix genotypes to some extent. For *in vitro* experimental evolution purposes, structure can be imposed by the addition of a matrix material to the growth medium.

## SUMMARY AND CONCLUSIONS

On the basis of the power of evolution to optimize molecular mechanisms, the successes of experimental evolution of microorganisms, and the fact that experimental evolution of antibiotic production in *Actinobacteria* has been achieved (7), we have here advocated experimental evolution of antibiotics as a platform for drug development. We have discussed evolutionary theory of critical importance, described a concrete procedure (Box 1), and addressed potential problems (Box 2).

Clearly, we have only outlined a first sketch of the approach, and much work remains. This is especially so on the empirical side, and it is to promote such work that we have written this paper. If an empirical foundation is established, however, relevant theory should also be developed further. Tasks that would benefit from theoretical work include the optimization of selection regimes to overcome whole suites of different resistance mechanisms with a single compound, as well as attaining a desirable antimicrobial spectrum. Moving beyond antibiotics, *Actinobacteria* also produce other medically important molecules, most notably, cytostatics used in the treatment of cancer. The application of experimental evolution to this context seems particularly challenging, as the high degree of similarity between cancer cells and healthy bystanders decreases the chances that a compound that is toxic to the former is not also toxic to the latter. The chances may by increased with a more complex evolution protocol that combines selection for toxicity to malignant cells and nontoxicity to healthy cells.

In conclusion, we have argued that evolution—the process that gives rise to both antibiotics and the resistance to them—can be leveraged to address the resistance problem currently experienced in health care systems around the world. Specifically, we make the case for experimental evolution in the laboratory to take control of the arms race between pathogens and drug development, in which pathogens currently reign alone.

BOX 1 ―SKELETON PROTOCOL

Here, we sketch a protocol to provide a concrete illustration of the approach. The procedure is represented in Fig. 2.

Material

The key materials are as follows:

1. An antibiotic-producing microorganism that is culturable and has a genome that can give rise to relevant genetic and phenotypic variation (e.g., an actinobacterium, whose genome contains a large variety of biosynthetic gene clusters).
2. A target microorganism that has a relevant resistance mechanism and can interact antagonistically with the producer. For the procedure we sketch below, the target organism should be fluorescently labeled, and it should be heat killed at a lower temperature than the producer (either naturally or by genetic manipulation).
3. A medium that is conducive to the antagonistic interactions between the producer and target organisms and provides adequate resistance to diffusion of cells and compounds to allow evolution of public goods (e.g., antibiotics). For the procedure we sketch below, the matrix material that provides the diffusion resistance should liquefy at a temperature that is lower than the heat killing temperature of the producer organism and solidify below the killing temperature of the target.

*Serial passage*

1. The producer organism is preevolved without the target organism or with a sensitive target. This allows the producer to adapt to the medium, possible mutagen, passage procedure, etc. It should therefore be performed exactly as in points 2 and 3, only with the exclusion of the target or its resistance mechanism.
2. The producer and target bacteria are cocultured in the medium. Either both bacteria are added at the same time, or one is allowed to establish before the other is added. In each growth cycle, fluorescence is measured to assess the extent to which the target bacterium is killed or inhibited in each replicate.
3. The culture is heated to liquefy the medium and kill the target bacterium (but not the producer), and agitated to break up cell clusters and mix the producer population. An aliquot of the culture, containing live producer or spores, is transferred to fresh medium, and mixed with fresh target bacteria. This initiates a new cycle at point 2.
4. When fluorescence readings indicate increased target killing, relevant samples are analyzed. For example, the MIDs (maximum inhibitory dilutions) or ED_50_s (50% effective dilutions; the dilutions that prevent 50% of growth or kill 50% of bacteria) of supernatants from evolved and nonevolved producers may be determined for the resistant target and a sensitive control strain, to distinguish improved potency of the product or product mixture from increased production.

Comments

The use of spatial structure creates a spatial association between an improved or novel compound and producer cells that share the underlying genotype. The transfer procedure with breaking of bacterial clusters and mixing of cells results in global scale competition among producers within replicates. Together, these two aspects promote the evolution of public goods (21), such as antibiotics and antiresistance compounds. It is important, however, that each round of culture be long enough to allow the benefits of compound production to accrue, lest the disruption of spatial structure in passages have a negative effect on selection for public goods production. (See Westhoff et al. [27].) As the optimal values for the diffusion resistance (for cells, nutrients, and compounds) and nutrient concentrations of the medium, the densities of producer and target, and the degree of resistance of the target cannot be precisely determined *a priori*, and evolution has a stochastic component, it is advisable to run the evolution experiment in a high-throughput manner with different levels of the above-mentioned parameters, as well as a large number of replicates. The level of resistance can be varied by genetic manipulation of the target organism or the addition of an antiresistance compound (e.g., a β-lactamase inhibitor), as relevant.

BOX 2 ―POTENTIAL PROBLEMS AND SUGGESTED SOLUTIONS

Here we, on theoretical grounds, discuss potential problems and suggest solutions with special emphasis on the protocol skeleton in Box 1.

1. *The selection pressure for overcoming the target’s resistance mechanism is swamped by selection for adapting to the growth medium, etc.* This is what the preevolution (point 1) in the protocol in Box 1 is aimed to address.
2. *The target organism evolves to survive transfers and then coevolves with the producer*. Depending on the extent of the problem, whether affected replicates show interesting results, and practical considerations, affected samples may be treated with, e.g., a cocktail of antibiotics to which the target—but not the producer—is sensitive, left as they are, or discarded.
3. *The timing of antibiotic production is suboptimal for relieving competition.* This may itself evolve to the better. It can also be addressed when the producer strain is chosen. For strains in which production is induced by nutrient limitation (a common feature), nutrient competition with the target is expected to promote production.
4. *The size of the producer population has conflicting effects.* Increasing producer population size increases the efficacy of selection (and decreases the effect of drift). It also widens the range of variation available for selection to act upon, since a larger number of cells provides more opportunity for relevant mutations to occur. However, within a limited culture volume, increasing population size increases population density. This weakens the link between the benefit conferred by, e.g., an improved compound and the genotype that gives rise to it, because at a higher population density, cells with other genotypes are closer, and therefore more likely to share the benefit of the improvement, despite not encoding it. This trade-off can be alleviated by the use of larger culture volumes. On the other hand, smaller volumes are more conducive to high-throughput methods.
5. *The competition with the target bacterium*, *and thus the selection pressure on the producer*, *is too strong or weak.* The bacteria and medium should be chosen so as to create antagonistic interaction, e.g., competition. This may be easier if the producer and target are ecologically similar. Relevant resistance can be achieved by genetic manipulation. As the killing efficiency increases during the evolutionary process, competition grows weaker. The strength of competition can be adjusted by using different target inoculum sizes for different sets of replicates or in different phases of the process. The degree of resistance can also be varied (see Box 1).
6. *Genetic variation is depleted.* Strong selection and genetic bottlenecks in culture transfers deplete genetic variation which, in turn, limits the scope for further evolution. Therefore, selection should not be too strong, and bottlenecks should not be too narrow. Given limited culture volume, the benefit of wide bottlenecks may trade off against the need to provide enough room for the population to expand under selection in each growth cycle. If necessary, a mutagen or mutator strain can be used to ensure that adequate levels of variation are maintained throughout the process.
7. *The evolved compound kills the target but is unsuitable as a drug for other reasons.* It seems implausible that this problem could be circumvented altogether. Some sources of problems may be possible to address by making the experimental evolution environment as similar as practically possible to the *in vivo* context in which the compound should be used. It is worth noting that the Waksman platform should be subject to this problem, but nonetheless, it was incredibly successful (2).
8. *The chemical space that can be reached by evolution has already been explored in drug discovery programs.* Given the difference between natural products and medicinal chemistry compounds (28) and, plausibly, between the fitness landscapes for antimicrobial natural products evolving in nature and under specific resistance-induced selection in the laboratory, this seems unlikely. In addition, it is estimated that only a small fraction of natural product antimicrobial compounds have been explored (14).
9. *Evolution is too slow.* The natural products in current use may have evolved over millions of years, and useful evolution on reasonable time scales may not be feasible. However, a similar argument applies to the evolution of antibiotic resistance in nature, but in the health care setting, it has occurred over years, and in experimental evolution *in vitro* over weeks (29). In addition, the evolution of antibiotic production from a previously silent pathway has been achieved by experimental evolution of an actinomycete for 4 months (7).
10. *The evolutionary response is not useful.* In addition to the evolution of novelty or modification, increased target killing can be achieved by a mere increase in the production of the original compound. However, both theory and experimental results indicate that the level of production should evolve to an optimum (24), where it should remain constant until other changes occur. The producer may also escape competition with the target by evolving to be less dependent on a limiting nutrient competed over. This may be addressed by inclusion of a sensitive target during the preevolution phase to saturate this potential, if possible. In addition, the serial passage procedure may favor mutants that have increased rates of growth (or sporulation, depending on the exact protocol) for any reason. Consequently, a large number of replicates is advisable to maximize the chances of useful evolutionary responses. Further analyses (see Box 1) can separate different kinds of responses. It is also worth noting that killing or inhibition of the competitor to increase nutrient availability is expected to be selected for even if other adaptations, such as increased growth rate, evolve first.
11. *Production of the original compound is turned off.* If there is a cost of compound production, and the target organism is so highly resistant to the original antibiotic that production of it does not pay off for the producer, selection disfavors production. This can lead to silencing of the pathway. If so, the compound cannot be improved by selection (because genetic variation in the pathway is not phenotypically manifest in a produced compound). The evolution of any antiresistance compounds would also be compromised, as these are efficacious only in conjunction with an antibiotic of the right type. This can be mitigated by optimizing the degree of resistance (see Box 1). A similar issue may occur during the preevolution phase, when selection for compound production is lacking. This trades off against the benefits of preevolution. If the goal is not to modify an existing compound, but to turn on a silent pathway, this should not be a problem.
12. *The structured medium limits oxygen availability*. A medium designed to limit the diffusion of cells and antibiotic compounds would also decrease the access of oxygen from the air to cells far away from the medium’s surface, a potential problem for strictly aerobic bacteria, such as streptomycetes. One approach would be to make the medium porous. Another is to accept that growth is confined to the volume close to the surface. The main drawback is that this constrains the useful volume of medium, especially if the procedure is to be run in a microwell format.

## References

[1] Centers for Disease Control and Prevention. 2013 Antibiotic resistance threats in the United States, 2013. Centers for Disease Control and Prevention, Atlanta, GA.

[2] Lewis K 2013 Platforms for antibiotic discovery. Nat Rev Drug Discov 12:371–387. doi:10.1038/nrd3975. 23629505

[3] Clardy J, Fischbach MA, Walsh CT 2006 New antibiotics from bacterial natural products. Nat Biotechnol 24:1541–1550. doi:10.1038/nbt1266. 17160060

[4] Brown ED, Wright GD 2016 Antibacterial drug discovery in the resistance era. Nature 529:336–343. doi:10.1038/nature17042. 26791724

[5] Fischbach MA, Walsh CT 2009 Antibiotics for emerging pathogens. Science 325:1089–1094. doi:10.1126/science.1176667. 19713519 PMC2802854

[6] Ling LL, Schneider T, Peoples AJ, Spoering AL, Engels I, Conlon BP, Mueller A, Schäberle TF, Hughes DE, Epstein S, Jones M, Lazarides L, Steadman VA, Cohen DR, Felix CR, Fetterman KA, Millett WP, Nitti AG, Zullo AM, Chen C, Lewis K 2015 A new antibiotic kills pathogens without detectable resistance. Nature 517:455–459. doi:10.1038/nature14098. 25561178 PMC7414797

[7] Charusanti P, Fong NL, Nagarajan H, Pereira AR, Li HJ, Abate EA, Su Y, Gerwick WH, Palsson BO 2012 Exploiting adaptive laboratory evolution of Streptomyces clavuligerus for antibiotic discovery and overproduction. PLoS One 7:e33727. doi:10.1371/journal.pone.0033727. 22470465 PMC3312335

[8] Baltz RH 2008 Renaissance in antibacterial discovery from actinomycetes. Curr Opin Pharmacol 8:557–563. doi:10.1016/j.coph.2008.04.008. 18524678

[9] Craig W, Andes D 2015 Cephalosporins, p 278–292.e4. *In* Mandell, Douglas, and Bennett’s principles and practice of infectious diseases. Elsevier, New York, NY.

[10] Fischbach MA, Walsh CT, Clardy J 2008 The evolution of gene collectives: how natural selection drives chemical innovation. Proc Natl Acad Sci U S A 105:4601–4608. doi:10.1073/pnas.0709132105. 18216259 PMC2290807

[11] Vingadassalon A, Lorieux F, Juguet M, Le Goff G, Gerbaud C, Pernodet J-L, Lautru S 2015 Natural combinatorial biosynthesis involving two clusters for the synthesis of three pyrrolamides in Streptomyces netropsis. ACS Chem Biol 10:601–610. doi:10.1021/cb500652n. 25415678

[12] Shi Y-M, Brachmann AO, Westphalen MA, Neubacher N, Tobias NJ, Bode HB 2019 Dual phenazine gene clusters enable diversification during biosynthesis. Nat Chem Biol 15:331–339. doi:10.1038/s41589-019-0246-1. 30886436

[13] Hoskisson PA, Fernández-Martínez LT 2018 Regulation of specialised metabolites in Actinobacteria – expanding the paradigms. Environ Microbiol Rep 10:231–238. doi:10.1111/1758-2229.12629. 29457705 PMC6001450

[14] Watve MG, Tickoo R, Jog MM, Bhole BD 2001 How many antibiotics are produced by the genus Streptomyces? Arch Microbiol 176:386–390. doi:10.1007/s002030100345. 11702082

[15] Davis AM, Plowright AT, Valeur E 2017 Directing evolution: the next revolution in drug discovery? Nat Rev Drug Discov 16:681–698. doi:10.1038/nrd.2017.146. 28935911

[16] Ohta T 1992 The nearly neutral theory of molecular evolution. Annu Rev Ecol Syst 23:263–286. doi:10.1146/annurev.es.23.110192.001403.

[17] Jansen G, Barbosa C, Schulenburg H 2013 Experimental evolution as an efficient tool to dissect adaptive paths to antibiotic resistance. Drug Resist Updat 16:96–107. doi:10.1016/j.drup.2014.02.002. 24594007

[18] Jensen PR 2016 Natural products and the gene cluster revolution. Trends Microbiol 24:968–977. doi:10.1016/j.tim.2016.07.006. 27491886 PMC5123934

[19] Schäberle TF, Lohr F, Schmitz A, König GM 2014 Antibiotics from myxobacteria. Nat Prod Rep 31:953–972. doi:10.1039/c4np00011k. 24841474

[20] Herrman J, Abou Fayad A, Müller R 2017 Natural products from myxobacteria: novel metabolites and bioactivities. Nat Prod Rep 34:135–160. doi:10.1039/c6np00106h. 27907217

[21] Griffin AS, West SA, Buckling A 2004 Cooperation and competition in pathogenic bacteria. Nature 430:1024–1027. doi:10.1038/nature02744. 15329720

[22] Allen RC, McNally L, Popat R, Brown SP 2016 Quorum sensing protects bacterial co-operation from exploitation by cheats. ISME J 10:1706–1716. doi:10.1038/ismej.2015.232. 26744811 PMC4918439

[23] Nogueira T, Rankin DJ, Touchon M, Taddei F, Brown SP, Rocha EPC 2009 Horizontal gene transfer of the secretome drives the evolution of bacterial cooperation and virulence. Curr Biol 19:1683–1691. doi:10.1016/j.cub.2009.08.056. 19800234 PMC2773837

[24] Gerardin Y, Springer M, Kishony R 2016 A competitive trade-off limits the selective advantage of increased antibiotic production. Nat Microbiol 1:16175. doi:10.1038/nmicrobiol.2016.175. 27668360 PMC5046839

[25] Kümmerli R, Griffin AS, West SA, Buckling A, Harrison F 2009 Viscous medium promotes cooperation in the pathogenic bacterium Pseudomonas aeruginosa. Proc Biol Sci 276:3531–3538. doi:10.1098/rspb.2009.0861. 19605393 PMC2817189

[26] West SA, Griffin AS, Gardner A, Diggle SP 2006 Social evolution theory for microorganisms. Nat Rev Microbiol 4:597–607. doi:10.1038/nrmicro1461. 16845430

[27] Westhoff S, Otto SB, Swinkels A, Bode B, van Wezel GP, Rozen DE 2019 Spatial structure increases the benefits of antibiotic production in Streptomyces. Evolution doi:10.1111/evo.13817. PMC6973283 31393002

[28] Rosén J, Gottfries J, Muresan S, Backlund A, Oprea TI 2009 Novel chemical space exploration via natural products. J Med Chem 52:1953–1962. doi:10.1021/jm801514w. 19265440 PMC2696019

[29] Toprak E, Veres A, Michel J-B, Chait R, Hartl DL, Kishony R 2011 Evolutionary paths to antibiotic resistance under dynamically sustained drug selection. Nat Genet 44:101–105. doi:10.1038/ng.1034. 22179135 PMC3534735

